# Hybrid ANFIS–MPA and FFNN–MPA Models for Bitcoin Price Forecasting

**DOI:** 10.3390/biomimetics10120827

**Published:** 2025-12-10

**Authors:** Ceren Baştemur Kaya, Ebubekir Kaya, Eyüp Sıramkaya

**Affiliations:** 1 Department of Computer Technologies, Nevşehir Vocational School, Nevşehir Hacı Bektaş Veli University, Nevşehir 50100, Türkiye; 2 Department of Computer Engineering, Engineering Architecture Faculty, Nevşehir Hacı Bektaş Veli University, Nevşehir 50100, Türkiye; ebubekir@nevsehir.edu.tr (E.K.); eyup@nevsehir.edu.tr (E.S.); 3 CEKA Software R&D Co., Ltd., Nevşehir 50100, Türkiye

**Keywords:** marine predators algorithm, adaptive neuro-fuzzy inference system, feed-forward neural network, hybrid optimization, metaheuristic algorithms, swarm intelligence, bitcoin price forecasting, time series analysis

## Abstract

This study introduces two hybrid forecasting models that integrate the Marine Predators Algorithm (MPA) with Adaptive Neuro-Fuzzy Inference Systems (ANFIS) and Feed-Forward Neural Networks (FFNN) for short-term Bitcoin price prediction. Daily Bitcoin data from 2022 were converted into supervised time-series structures with multiple input configurations. The proposed hybrid models were evaluated against six well-known metaheuristic algorithms commonly used for training intelligent forecasting systems. The results show that MPA consistently yields lower prediction errors, faster convergence, and more stable optimization behavior compared with alternative algorithms. Both ANFIS-MPA and FFNN-MPA maintained their advantage across all tested structures, demonstrating reliable performance under varying model complexities. All experiments were repeated multiple times, and the hybrid approaches exhibited low variance, indicating robust and reproducible behavior. Overall, the findings highlight the effectiveness of MPA as an optimizer for improving the predictive performance of neuro-fuzzy and neural network models in financial time-series forecasting.

## 1. Introduction

In recent years, many investors have turned to cryptocurrency markets alongside traditional instruments, such as stocks. The growing adoption of blockchain technology has played a significant role in this shift. The ability to track transactions transparently on the blockchain has strengthened investor confidence, while features such as traceability, resistance to tampering, and difficulty of counterfeiting have increased the appeal of cryptocurrencies [1]. Bitcoin is the first cryptocurrency and was introduced in 2009. It is not regulated or overseen by any central authority; in other words, it operates with a fully decentralized structure [1,2,3]. Cryptocurrencies are inherently limited in supply, and the number of Bitcoins in circulation is capped. Bitcoin relies on strong cryptographic algorithms for its security, and it continues to operate as an independent investment asset, separate from traditional commodities [4]. The demand for Bitcoin has been rising steadily because of these characteristics. However, cryptocurrency prices tend to fluctuate significantly, influenced by factors such as ease of trading and their limited supply. As a result, they often display highly unstable behavior [4,5]. This situation makes it important to predict the Bitcoin price in order to make the right investment.

After an examination of the literature, it was observed that artificial intelligence techniques such as ANNs and ANFIS are used in cryptocurrency price prediction. ANNs are an artificial intelligence technique that can be used in many areas, such as learning, association, and optimization. It produces an output in response to the inputs presented to it [6,7]. There have been previous studies on ANNs and cryptocurrencies [2,3].

Nakano et al. [8] developed an ANN-based model to forecast Bitcoin’s daily trading activity. The input time series was constructed using 15 min interval data, and the authors reported that the proposed model achieved strong predictive performance. Radityoet et al. [4] compared four ANN-based approaches—BPNN, a genetic algorithm neural network, a genetic algorithm BPNN, and neuro-evolution of augmenting topologies—to predict Bitcoin’s market value. Their results indicated that the standard BPNN model outperformed the other methods. Sin and Wang [9] employed a selective neural network ensemble optimized with a genetic algorithm to investigate the relationship between Bitcoin’s next-day price movements and various Bitcoin properties. Their findings showed that the proposed ensemble model achieved strong predictive performance. Aghashahi and Bamdad [10] utilized ANNs such as Feedforwardnet, Fitnet, and Cascade networks to estimate the future price of Bitcoin. They indicated that the Fitnet network was more successful. Pagnottoni [11] developed an ANN-based pricing model for Bitcoin. The study reported that this model outperformed traditional approaches in estimating Bitcoin prices. Othman et al. [12] developed an ANN-based model using the RapidMiner platform to predict Bitcoin prices. Their study incorporated open, high, low, and close values as inputs, and the results showed that the proposed model performed well in forecasting both Bitcoin and other cryptocurrency prices. Abbasi et al. [13] compared a deep learning-based dual-stage Partial Least Squares–Structural Equation Modeling (PLS-SEM) combined with ANN to the traditional single-stage PLS-SEM approach for cryptocurrency prediction. Their findings showed that the dual-stage PLS-SEM and ANN model produced more accurate and reliable results. Jang and Lee [14] used a Bayesian neural network model to analyze the time series behavior of Bitcoin prices. They first selected Blockchain-related variables that influence Bitcoin’s supply–demand dynamics and used these features to train the model. The performance of the proposed approach was evaluated against both linear and nonlinear benchmark models, and the results showed that the Bayesian neural network achieved superior predictive accuracy. Sujatha et al. [15] trained an ANN by using Levenberg–Marquardt, Bayesian Regularization, and Scaled Conjugate Gradient algorithms on Bitcoin trend analysis. When the error histogram and regression plots results were examined, it was reported that the Bayesian Regularized Neural Network had a more successful forecast. Jay et al. [5] proposed a stochastic neural network model for predicting the cryptocurrency price. They trained the MLP and LSTM models for Bitcoin, Ethereum, and Litecoin. They indicated that the proposed model was more successful than the deterministic models. Sohaib et al. [16] utilized ANN and different methods for measuring the relation between the technology acceptance-based dimensions and the intention to use cryptocurrency, such as Bitcoin. Uras et al. [17] employed two neural network models—MLP and LSTM—along with various statistical and machine learning techniques to predict the daily closing prices of Bitcoin, Litecoin, and Ethereum using historical price and volume data. Their results indicated that the proposed models achieved strong predictive performance. Cahyani and Nuha [18] developed a system by using the backpropagation ANN and Weka software to minimize hacking attacks on cryptocurrencies. Hansun et al. [19] applied several approaches, including three recurrent neural network architectures—LSTM, bidirectional LSTM, and GRU—to estimate the values of various cryptocurrencies. Their study focused on Bitcoin, Ethereum, Cardano, Tether, and Binance Coin. Kaya [20] proposed a model based on the FPA and ANN for predicting Bitcoin price. Time series analysis was realized by using daily Bitcoin data. It has been indicated that the proposed model was successful.

ANFIS is a hybrid method consisting of a combination of ANNs and a fuzzy inference system [21]. After an examination of the literature, it was observed that there were studies conducted with ANFIS on cryptocurrencies [22,23,24,25,26,27]. Ergün and Karabıyık [27] employed the neuro-fuzzy controller forecasting system (PATSOS) to predict Monero prices and trends. The PATSOS framework consists of two ANFIS-based subsystems, CON-ANFIS and PR-ANFIS. Their study reported that the proposed system produced effective forecasting results. Jaber et al. [24] proposed a forecasting model that integrates ANFIS, Haar mathematical functions, and a nonlinear spectral approach based on the maximum overlapping discrete wavelet transform (MODWT), using daily Bitcoin closing prices. They compared their hybrid model with both the ARIMA model and a standard ANFIS model, and reported that the proposed approach achieved superior predictive performance. Birim and Sönmez [25] utilized neuro-fuzzy systems to forecast the daily price of Ethereum, Ripple, and Litecoin by using a combination of Twitter sentiment and Google trend data. Atsalakis et al. [23] employed the PATSOS hybrid neuro-fuzzy controller to forecast the direction of daily Bitcoin price changes. They compared the proposed approach with two alternative computational-intelligence models: one developed using a simpler neuro-fuzzy structure and another based on an ANN. Their results showed that the PATSOS model delivered more effective forecasting performance. Karabıyık and Ergün [26] employed the ANFIS model to predict the Bitcoin price. They reported that the results were consistent with the real data. Birim and Sonmez [22] trained ANFIS by using the PSO to estimate cryptocurrency return rates. They reported that the proposed model gave successful results.

Achieving reliable results with ANNs and ANFIS requires carefully managed training procedures, as the choice of optimization algorithm directly influences model accuracy. The MPA is a recently developed metaheuristic that has shown strong performance across various optimization problems. Although only a limited number of studies have investigated the use of MPA for training ANNs or ANFIS [28,29,30], its application to cryptocurrency forecasting remains largely unexplored. In this study, MPA is used to train both ANFIS and FFNN models, leading to two hybrid forecasting approaches. The performance of MPA is then compared with several established metaheuristic algorithms, including ABC, FPA, DA, RIME, TLBO, and MVO, under identical experimental conditions. This limited presence of MPA-based training in the literature underscores the novelty and motivation behind the proposed framework.

In line with these motivations, the research plan of this study is structured as follows. First, two hybrid forecasting models are constructed by integrating the MPA with ANFIS and FFNN. Second, daily Bitcoin data for 2022 are transformed into supervised time-series structures with different input configurations (S1–S3). Third, each model is trained using MPA, and its performance is compared across varying membership function sizes (for ANFIS) and network structures (for FFNN). Finally, the effectiveness of MPA is evaluated against several well-known metaheuristic algorithms—ABC, FPA, DA, MVO, RIME, and TLBO—under identical experimental settings. This framework outlines the methodological flow of the study and clarifies the steps taken to assess the proposed approach.

The aim of this study is to examine the performance of the MPA in training ANFIS and FFNN models. Therefore, the comparison framework was intentionally limited to metaheuristic algorithms with similar optimization roles (ABC, FPA, DA, MVO, RIME, TLBO), ensuring that all methods solve the same training problem under identical conditions. Deep learning architectures such as LSTM or Transformer involve fundamentally different model structures and training mechanisms; including them would shift the focus from optimizer-level evaluation to architecture-level comparison, which was not the objective of this work.

## 2. Materials and Methods

### 2.1. Marine Predators Algorithm (MPA)

**Figure 1:**
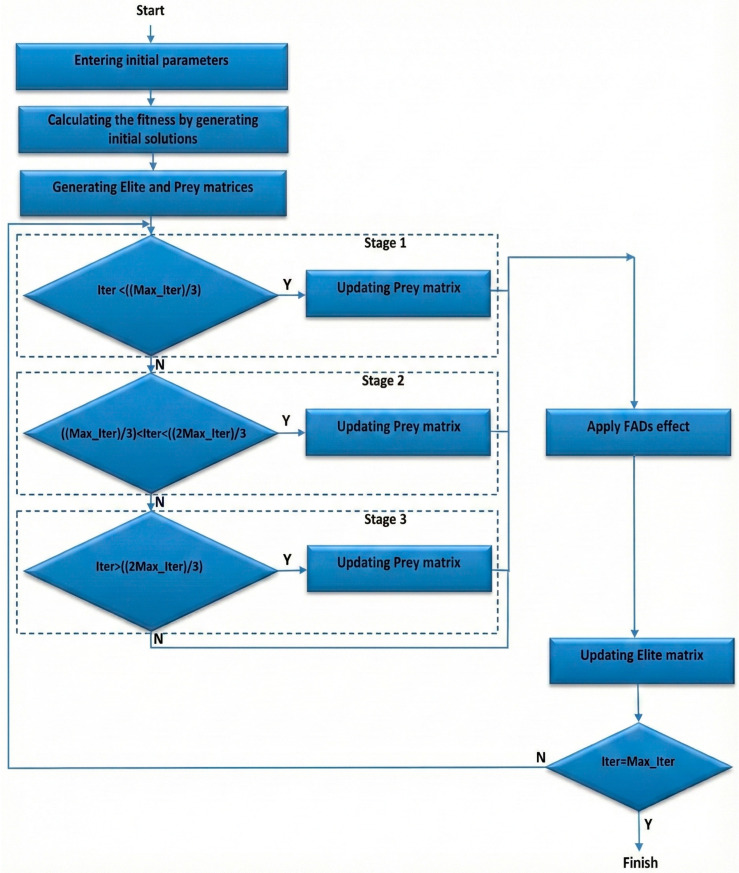
Flowchart showing the optimization process of MPA.

MPA developed by Faramazi et al. [31] is a swarm-based meta-heuristic algorithm inspired by foraging strategies between prey and predator in the ocean. Lévy and Brownian movements are used as foraging strategies in MPA. Brownian movement is preferred in cases of high prey density, whereas Lévy movement is preferred in cases where prey density is low. In addition, the speed rate (v) of predator to prey is important in MPA. The optimization process is carried out with different scenarios according to the speed rate. The optimization process of MPA is shown by the flowchart presented in Figure 1.

When the flowchart presented in Figure 1 is examined, it can be seen that during the optimization process, the preliminary solution is first spread into the search space. Afterwards, the Elite and Prey matrices are created. In the Elite matrix, which checks searching and finding of prey, the best solution is assigned as the best predator. Predators update their position according to the Prey matrix. After these steps, it moves on to the stages created by taking into consideration v. At the high-speed rate, stage 1 is carried out, and the Prey matrix is updated according to the Stage 1 rules. Stage 2 occurs when the unit speed rate and the Prey matrix are updated by applying the stage 2 rules. Stage 3 occurs at a low-speed rate, and the Prey matrix is updated according to the stage 3 rules. After these stages, the FAD effect rules are applied, which include factors such as Fish Aggregating Devices or eddies that influence predator behavior. In the final step, the solutions are evaluated. If the solutions are better than the previous one, the Elite matrix is updated.

### 2.2. Adaptive Neuro Fuzzy Inference System (ANFIS)

**Figure 2:**
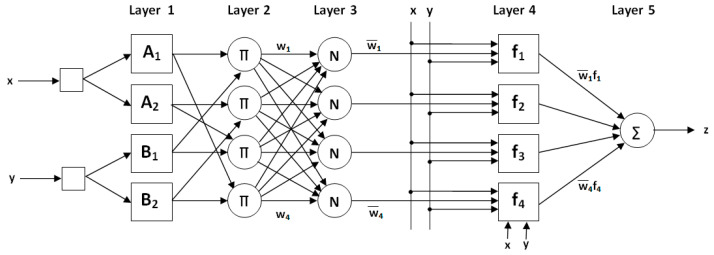
An example of an ANFIS structure with two inputs and one output.

ANFIS is an artificial intelligence technique achieved with ANN and a fuzzy inference system [21]. Features of the ANN, such as learning, classification, association, and the ability to produce results even with incomplete information, are important. However, ANNs’ behavior cannot be explained. In other words, it is not known how and why the produced solution was formed. The fuzzy inference system has no learning ability. However, it works according to the If-Then rule. Thus, the relationship between input and output can be explained. The advantages of these two systems formed the basis of ANFIS [21]. An example of an ANFIS structure is presented in Figure 2.

As seen in Figure 2, ANFIS consists of five layers. In layer 1, named the fuzzification layer, fuzzy clusters are obtained from the inputs by using membership functions that can have different shapes and features. Additionally, membership degrees of MFs are calculated. In Layer 2, called the rule layer, the firing strengths ($w_{i}$) for each rule are calculated by using the membership degrees in Layer 1. In Layer 3, named the normalization layer, normalized firing levels ($\bar{w_{i}}$) are calculated by using $w_{i}$. In Layer 4, called the defuzzification layer, the output for each rule is obtained by multiplying $\bar{w_{i}}$ with a first-degree polynomial. In Layer 5, called the summation layer, the calculated outputs for all rules are summed, and the ANFIS output is obtained.

### 2.3. Feed Forward Neural Network (FFNN)

ANN is an artificial intelligence technique developed based on the characteristics of the human brain. It is a computer system that can learn like humans and produce new information as a result. ANNs consist of artificial neurons. Artificial neurons are connected to each other, and each connection has a value [6,7].

**Figure 3:**
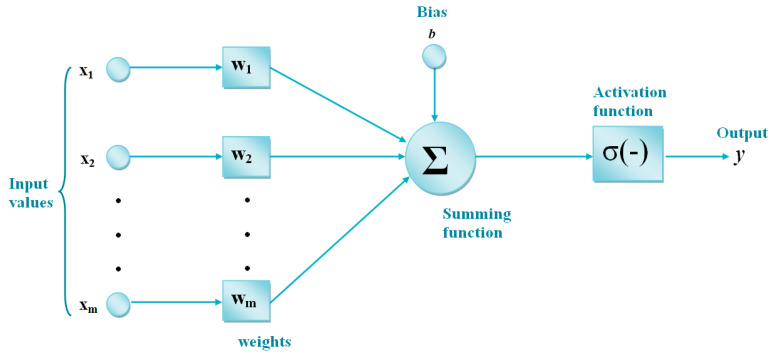
Feed-forward neural networks.

ANNs can be applied in subjects such as association, optimization, generalization, classification, and feature determination. Also, one of the most important advantages of ANNs is the learning feature. A training algorithm is needed for the learning process of ANNs. The weight values in the structure of the ANN are optimized by using this training algorithm [6,7]. In this study, FFNNs are used. In FFNNs, information transfer occurs from the input layer to the output layer, as shown in Figure 3. FFNNs consist of three layers: input, hidden, and output. Output data is obtained from the input data through the calculations occurring in the artificial neurons in these layers. ANNs use the training dataset in the learning process. In the test process, the test is performed on previously unknown data.

## 3. Simulation Results

In this study, two methods are proposed for the short-term prediction of Bitcoin prices. These are MPA-based ANFIS and MPA-based FFNN. Daily Bitcoin data from 2022 was used in the applications, particularly in performing the time-series analysis. In order to apply the relevant data to the ANFIS and FFNN, the data was converted into datasets consisting of inputs and outputs. Past data also sheds light on future data. For this reason, studies were carried out on three different systems, as seen in Table 1. In S1, price information belonging to the last two days was used to obtain the output. Price information belonging to the last three days was utilized in S2. In S3, price information belonging to the last four days was used. In other words, S1 consists of two inputs, and S2 and S3 consist of three and four inputs, respectively. S1, S2, and S3 all have an output. In all systems, the dataset consists of 360 data pairs in total. Of these, 288 were used in the training process. The remaining amount was reserved for the testing process. Bitcoin values in the dataset were scaled between 0 and 1 due to their large size. Throughout the study, analyses were performed on the scaled data. MSE was used in both ANFIS and FFNN training. The population size of the MPA was selected as 20. The maximum number of generations was 2500.

The hyperparameters used in this study were determined to be in line with commonly adopted practices in metaheuristic-based ANFIS and ANN training. The population size of MPA was set to 20, as several studies using MPA or comparable optimizers have reported that small-to-medium population sizes provide efficient convergence without imposing a high computational cost. The maximum number of generations was fixed at 2500. Increasing this limit further often slows the convergence rate and may not yield meaningful improvement in solution quality, a trend frequently noted in the optimization literature.

In this study, daily Bitcoin prices from 2022 were used. Even though data from many years are available, a single year already yields a sufficiently large set of training samples once the series is converted into multi-input structures (S1–S3). Using several years of data would produce a much larger input–output matrix, which in turn increases the number of parameters to be optimized in ANFIS as well as the size of the weight matrices in FFNN. This expansion leads to a noticeable rise in computational cost during metaheuristic training. Therefore, a one-year period was preferred to keep the optimization process manageable while still reflecting a period of strong market volatility.

At the same time, the models were constructed using a univariate time-series framework in which only past price values were used as inputs. The aim was to examine the predictive capability of ANFIS and FFNN when trained with MPA under the intrinsic nonlinear dynamics of Bitcoin price movements. Including exogenous variables, such as transaction volume, sentiment indicators, or technical metrics, would increase model dimensionality and introduce additional sources of variability, making it more difficult to isolate the effect of the optimization algorithm itself. Therefore, external variables were intentionally excluded in order to focus on the core time-series forecasting problem.

In many forecasting studies, cross-validation methods such as k-fold or rolling-window validation are widely used. However, applying these techniques in the context of metaheuristic training would require repeating the entire optimization process multiple times, which is computationally impractical for ANFIS and FFNN models trained with MPA. To ensure a fair and systematic separation of data while keeping the computational load manageable, a deterministic sampling rule was adopted. Specifically, each sample with index i was assigned to the test set if i mod 5 = 0; otherwise, it was included in the training set. This procedure distributes test samples uniformly throughout the dataset and provides a stable evaluation framework without introducing high computational cost.

**Figure 4:**
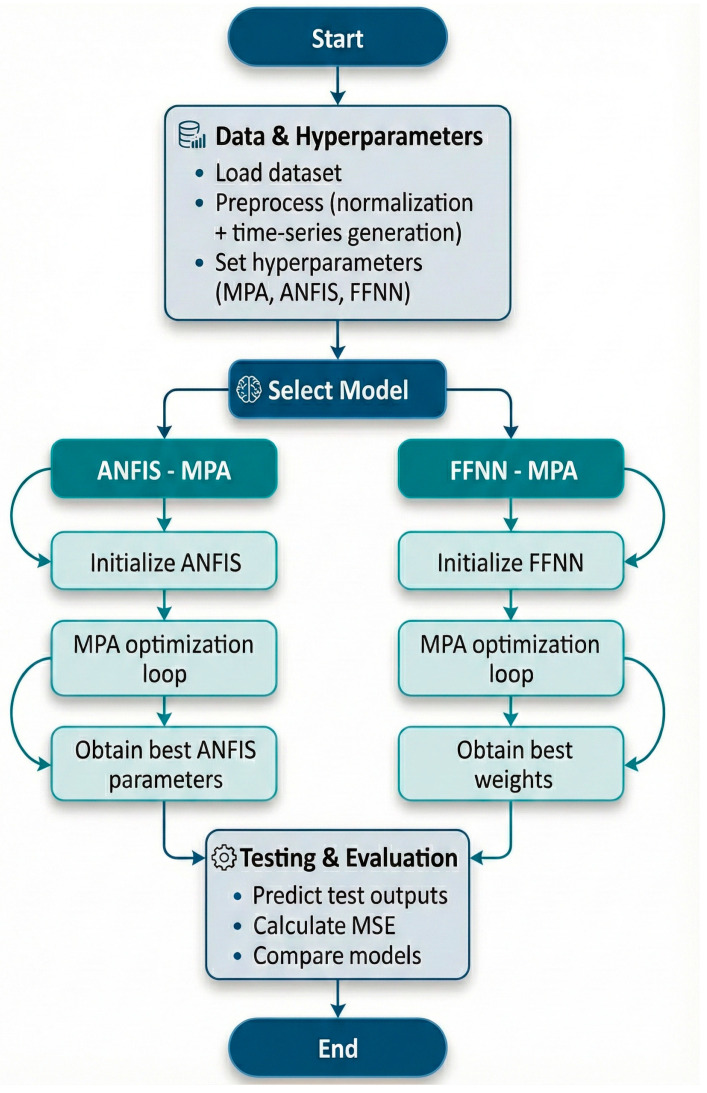
Overall workflow of the proposed hybrid forecasting framework.

Figure 4 illustrates the complete workflow followed in the proposed hybrid forecasting framework. The process starts with loading the Bitcoin dataset, followed by preprocessing steps such as normalization and supervised time-series generation. After defining the hyperparameters for both the MPA and the learning models, the workflow diverges into two alternative optimization paths: ANFIS–MPA and FFNN–MPA. In the ANFIS path, MPA optimizes the membership-function parameters and rule consequents, whereas in the FFNN path, it optimizes the network weights. Following optimization, the best-performing parameters obtained from each model are used to generate test predictions. Finally, MSE is calculated to evaluate forecasting performance. This workflow summarizes all major computational steps of the proposed hybrid approach in a clear and structured manner.

### 3.1. Training ANFIS Using MPA for Short-Term Forecast of Bitcoin

In this section, the performance of MPA-based ANFIS training for short-term Bitcoin price prediction is evaluated. The gbellmf was used in all configurations, as it has been shown to perform effectively in previous forecasting studies. To examine the impact of model complexity, different numbers of MFs were tested. For S1, 2, 3, and 4 MFs were evaluated. Since the number of inputs increases the size of the parameter search space, S2 was tested with 2 and 3 MFs, while S3 was limited to 2 MFs. This setup allowed for a balanced assessment of performance while keeping the optimization process computationally manageable.

**Figure 5:**
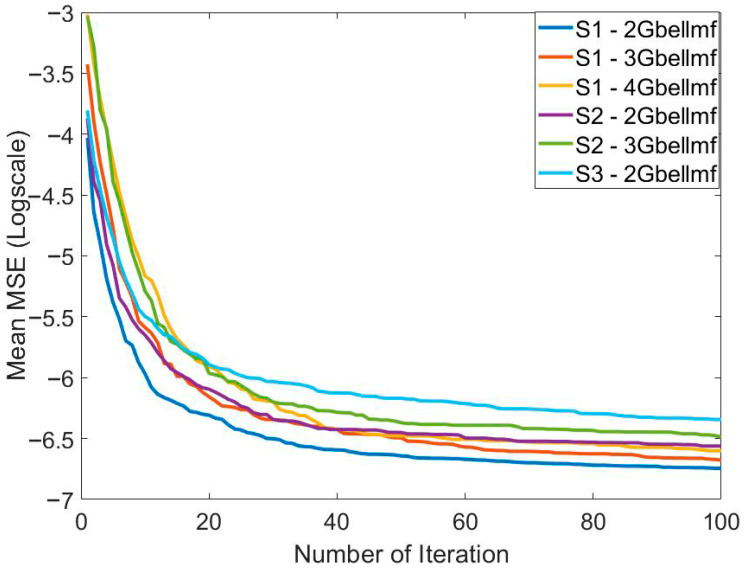
Comparison of the effect of the systems applied and the number of membership functions on the convergence to solve the relevant problem.

**Figure 6:**
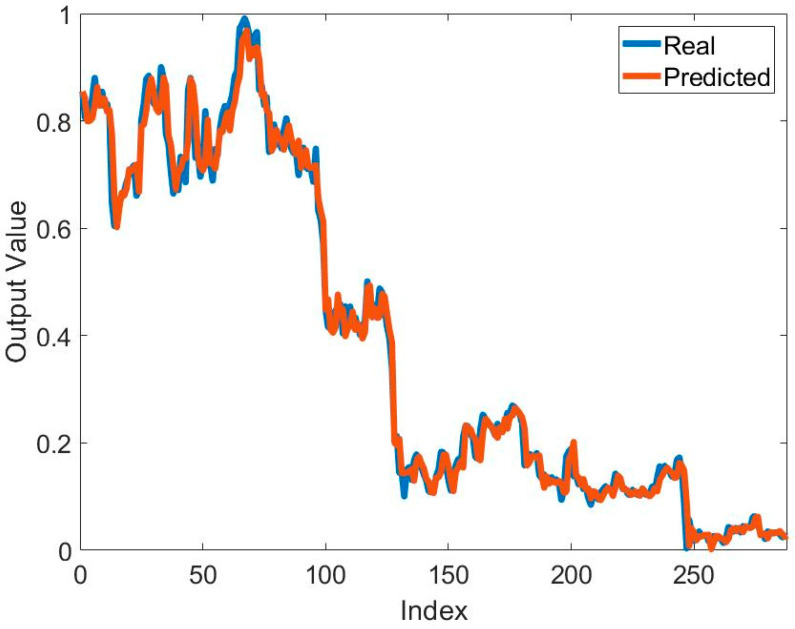
Comparison of the real output and predicted output obtained by using ANFIS and MPA to solve the relevant problem.

Table 2 presents the training results of the method based on the MPA and ANFIS for predicting the price of Bitcoin. It can be seen that the best and mean best training result for predicting Bitcoin price is obtained in S1. The best training error value is 7.8953 × 10^−4^. The best mean training error value is 9.4225 × 10^−4^. These results were obtained using 2MFs. It seems that the number of MFs affects the solution quality. In addition to solution quality, its effect on convergence speed is also important. Figure 5 shows the effect of the systems used and the number of MFs on convergence. In particular, the increase in the number of parameters to be optimized also reduced the convergence speed. The lowest number of parameters belongs to 2MFs of S1. Therefore, the fastest convergence belongs to this one. The highest number of parameters belongs to 2MFs of S3. Therefore, this model has the slowest convergence. The mean error values in all models are at the 10^−4^ level. Standard deviation values are at the 10^−5^ level. This shows that the training process was successful. To show the success of the training process, the graphs of the actual output and the predicted output are compared in Figure 6. In general, it is seen that the actual output and the predicted output overlap each other. This is one of the indicators of a successful training process.

Table 3 presents the results obtained with the MPA-based ANFIS method. As in the training results, the best mean error value in the testing process was found with 2 MFs on S1. This error value is 8.7879 × 10^−4^. Other mean error values found are 9 × 10^−4^ and above. As with the training results, the mean error values in all models are at the 10^−4^ level. Standard deviation values are at the 10^−5^ level. These results show that the testing process was as successful as the training process. Additionally, the best test error value was found to be 7.66741 × 10^−4^ in S3.

**Figure 7:**
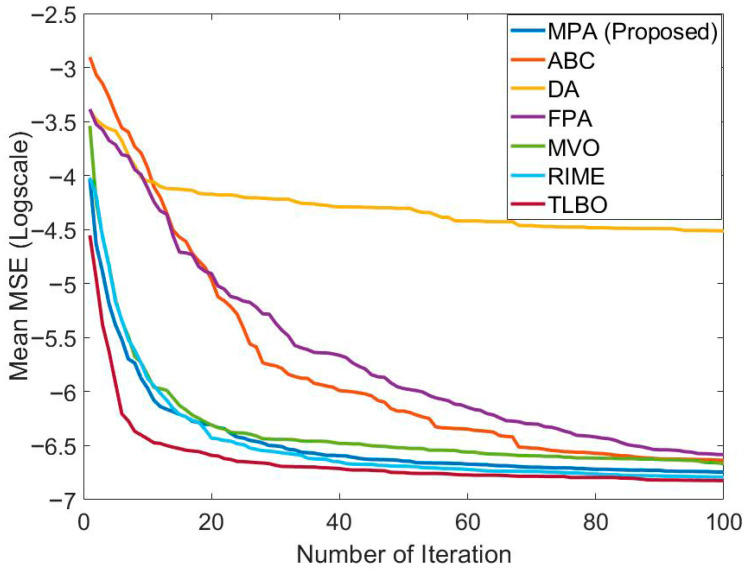
Comparison of the convergence speed of MPA with different algorithms in the ANFIS training carried out to solve the relevant problem.

In Table 4, the performance of MPA is compared with ABC, DA, FPA, MVO, RIME, and TLBO in the ANFIS training for short-term Bitcoin prediction. These results were obtained using S1 and 2MFs. The population size and maximum number of generations of all algorithms were taken as 20 and 2500, respectively. The best training and testing error was obtained with MPA. The training and testing errors found with MPA were 9.4225 × 10^−4^ and 8.7279 × 10^−4^, respectively. After MPA, TLBO had the best training and testing error value. The training error of TLBO is 9.71073 × 10^−4^. The test error of TLBO is 8.8361 × 10^−4^. The training error values of other algorithms are at the 10^−3^ level. Test error values of other algorithms vary between 8.96077 × 10^−4^ and 1.00396 × 10^−3^. After TLBO, the most effective algorithm in terms of test error value was RIME. The worst mean training and testing error values belonged to DA. Convergence graphs of the algorithms are presented in Figure 7. It can be observed that MPA, TLBO, and RIME have effective convergence graphs.

### 3.2. Training Feed-Forward Neural Network Using MPA for Short-Term Forecast of Bitcoin

In this section, the performance of the MPA-based FFNN training for short-term Bitcoin price prediction is evaluated. For each of the systems (S1, S2, and S3), several representative network structures were tested by varying the number of neurons in the hidden layer (3, 6, 9, 12, 15, 18, and 21). This selection allowed for the evaluation of different model complexities without exhaustively testing all possible configurations, which would significantly increase training time due to the rapid growth in the number of trainable parameters. The sigmoid activation function was used, and bias terms were included in all models. The MPA control parameters were kept consistent with those used in the ANFIS experiments to ensure comparable optimization conditions.

**Figure 8:**
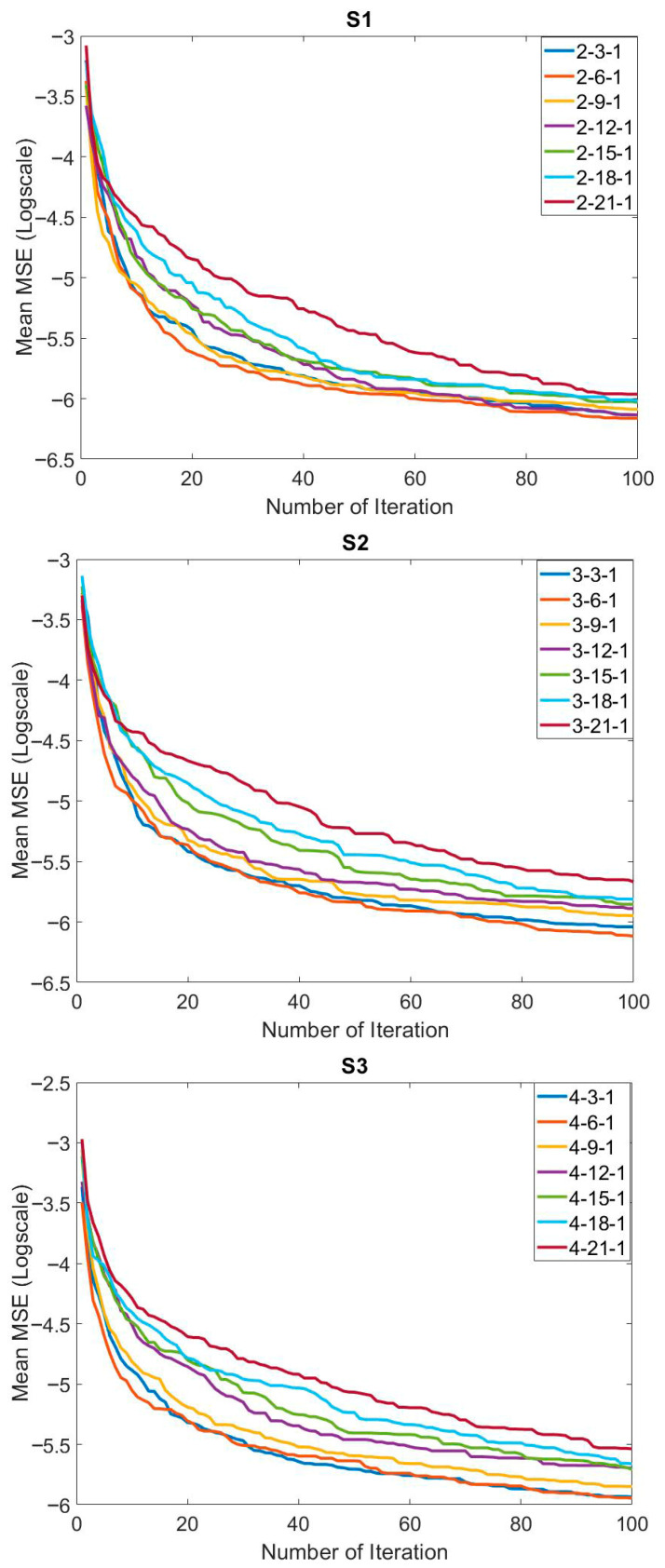
Comparison of the effect of the systems applied and the network structures on the convergence to solve the relevant problem.

**Figure 9:**
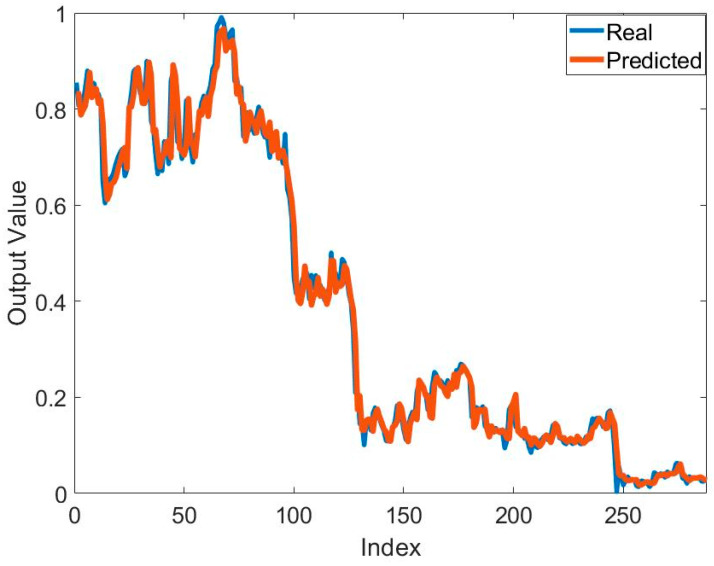
Comparison of the real output and predicted output obtained by using FFNN and MPA to solve the relevant problem.

The training results obtained with the MPA- and FFNN-based hybrid method to solve the relevant problem are presented in Table 5. In general, similar mean training error values were achieved in all systems and all network structures. The mean error value was found to be 1 × 10^−3^. The best training error value obtained was 9.6513 × 10^−4^ by using the 3-15-1 network structure on S2. The worst error values in all systems were at the 10^−3^ level. Standard deviation values were at the 10^−5^ levels. Although mean error values were generally close to each other in systems and network structures, convergence speeds were also important. A comparison of the convergence speeds of the network structures for S1, S2, and S3 is presented in Figure 8. The convergence speed of the 2-21-1 network structure, which has the highest number of neurons in S1, was the lowest, followed by the 2-18-1. Convergence was fast in networks with a low number of neurons in S1. Similar convergences were generally observed in the convergence graphs in S2 and S3. In particular, the large number of neurons increased the number of parameters to be optimized. This may be one of the important reasons for the low convergence speed. In order to concretely evaluate the success of the training process carried out with the proposed MPA and FFNN-based hybrid method, the graphs of the actual output and the predicted output are compared in Figure 9. In general, it is seen that the actual output and the predicted output overlapped each other. This is one of the indicators of a successful training process.

The testing results obtained with the MPA- and FFNN-based hybrid method to solve the relevant problem are presented in Table 6. Unlike the training results, the mean test error values spread over a wider range. Increasing the number of neurons in S1 and S2, up to a 2-15-1 network structure, contributed positively to the solution. The best mean error value was obtained by using the 2-15-1 network structure on S1. The best mean error value found was 8.72483 × 10^−4^. The best test error value was found by utilizing the 3-21-1 network structure on S2. The best test error value obtained was 8.05415 × 10^−4^. The mean error values in all system and network structures were better than the training results. According to the training results, the mean error values were at the 10^−3^ level, whereas the test results were at the 10^−4^ level.

**Figure 10:**
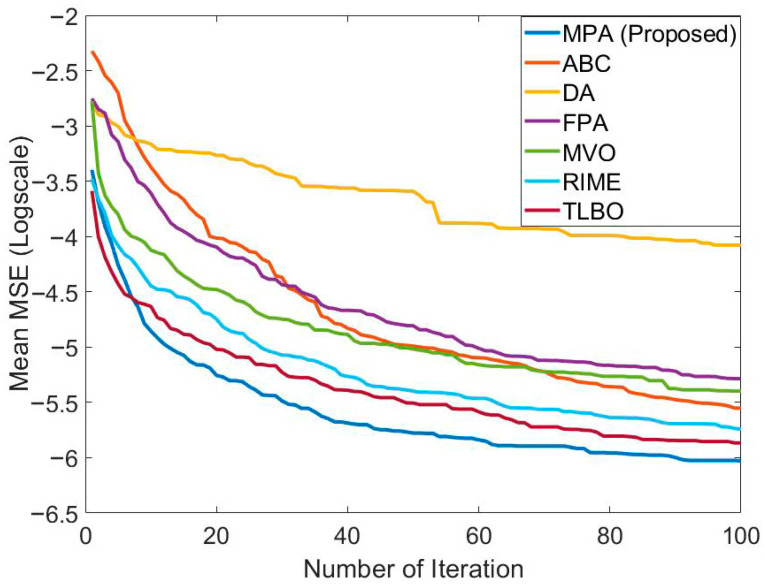
Comparison of the convergence speed of MPA with different algorithms in the FFNN training carried out to solve the relevant problem.

In Table 7, the performance of MPA is compared with ABC, DA, FPA, MVO, RIME, and TLBO in the FFNN training for short-term bitcoin prediction. The results for all algorithms were obtained using the 2-15-1 network structure on S1. The population size and maximum number of generations of all algorithms were taken as 20 and 2500, respectively. The best mean test error value was obtained with MPA, similarly to the training process. The mean training and testing error values obtained with MPA are 1.02777 × 10^−3^ and 8.72483 × 10^−4^, respectively. After MPA, the best mean training error value was found with MVO. After MPA, the best mean test error value was found with TLBO. The worst results in both training and testing were obtained with DA. Convergence graphs of the algorithms are presented in Figure 10. It seems that the best convergence was achieved with MPA. The three algorithms with the best convergence were MPA, TLBO, and RIME. This shows that MPA was effective in convergence as well as solution quality.

## 4. Discussion

In studies where ANFIS or neural networks are trained using population-based metaheuristic algorithms, the overall training time is highly dependent on the hardware configuration, programming environment, and the number of candidate solutions evaluated at each iteration. For this reason, similar works in the literature typically prioritize comparative indicators such as convergence curves and error metrics rather than absolute runtime. In this study, all algorithms were executed under the same computational settings, ensuring a fair comparison based on their optimization performance.

The superior performance of MPA in both ANFIS and FFNN training can be attributed to several characteristics of the algorithm. MPA uses a phase-based optimization strategy in which the search behavior shifts between Brownian and Lévy movements depending on the predator–prey velocity ratio. This mechanism allows the algorithm to maintain a balanced exploration–exploitation process throughout the optimization. Additionally, the elite matrix and the FAD-disruption component help the algorithm avoid premature convergence by periodically introducing controlled diversity. These features collectively enable MPA to converge faster while maintaining solution quality, which explains its stronger performance compared with the other metaheuristic algorithms evaluated in this study.

All experiments were repeated 30 times independently in order to account for the stochastic nature of metaheuristic optimization. The reported mean, best, worst, and standard deviation values summarize the distribution of the outcomes across the repeated runs. Although nonparametric statistical tests, such as Wilcoxon or Friedman, could be applied, including the full set of pairwise and multi-algorithm comparisons would considerably expand the scope of the results section. For clarity and consistency with similar metaheuristic forecasting studies, aggregated statistical indicators were used to present the comparative performance.

In this study, MSE was used as the primary evaluation metric, as it is the standard loss function minimized during the metaheuristic training of ANFIS and ANN models. Using multiple error measures, such as RMSE, MAE, or MAPE, would not alter the optimization procedure, since these metrics are monotonic functions of MSE and would, therefore, lead to identical model rankings. For this reason, and to maintain clarity in the comparative analysis, MSE was adopted as the sole performance indicator, which is consistent with many metaheuristic-based forecasting studies.

This study has several limitations that should be acknowledged. First, the experiments were conducted exclusively on Bitcoin, which exhibits unique volatility characteristics and may not fully represent the behavior of other cryptocurrencies or financial assets. Applying the proposed hybrid models to alternative cryptocurrencies—such as Ethereum, BNB, or Solana—or to traditional financial markets may reveal different levels of sensitivity to model structure, optimization dynamics, and data patterns. Second, the analysis was carried out using a univariate time-series framework. Including additional variables such as trading volume, sentiment indicators, or market indices may influence model performance in more complex market environments. Finally, the study focused on a one-year dataset to maintain computational feasibility. Extending the approach to multi-year datasets or diverse market regimes may provide deeper insights into the generalizability of the hybrid models.

## 5. Conclusions

Within the scope of this study, the performance of two MPA-based hybrid methods for short-term prediction of Bitcoin price was examined. These hybrid methods were ANFIS-MPA and FFNN-MPA. Detailed studies have previously been carried out for both methods. The method is especially aimed at predicting future Bitcoin prices using past prices. Different systems have been created for this. The effects of FFNN’s network structures and ANFIS’s membership function numbers on the performance were examined. However, the performance of MPA in training FFNN and ANFIS has been compared with meta-heuristic algorithms such as ABC, DA, FPA, MVO, RIME, and TLBO. The general conclusions of this study are as follows:

- Effective results have been achieved in both hybrid methods for the short-term prediction of Bitcoin price.
- The inputs used and the number of inputs affect the training and testing performance of the proposed hybrid methods.
- The best mean training and testing error found with ANFIS-MPA were 9.4225 × 10^−4^ and 8.7279 × 10^−4^, respectively.
- The mean training and testing error values obtained with FFNN-MPA were 1.02 × 10^−3^ and 8.72483 × 10^−4^, respectively.
- While ANFIS-MPA and FFNN-MPA had similar success in the testing processes, it was observed that ANFIS-MPA was more successful than FFNN-MPA in the training process.
- It was observed that the performance of MPA is better than ABC, DA, FPA, MVO, RIME, and TLBO in the ANFIS and FFNN training performed for the short-term prediction of Bitcoin prices.
- In addition to the solution quality of MPA, it was observed that the convergence speed of MPA is also effective in solving the relevant problem.
- In parallel with the training results, the test results were also successful in both hybrid methods.

Future research may extend the proposed hybrid models in several directions. Their robustness can be evaluated using multi-year datasets and different volatility regimes, and the framework may be applied to other cryptocurrencies or financial markets to examine their behavior across diverse market structures. Incorporating exogenous variables—such as transaction volume, market sentiment, or technical indicators—could provide additional insights into the influence of external factors on forecasting accuracy. Furthermore, conducting a formal sensitivity analysis of key hyperparameters, together with the use of broader evaluation metrics, may offer a deeper understanding of the optimization dynamics and the generalizability of the proposed models.

## Figures and Tables

**Table 1:** Systems created for Bitcoin prediction.

System	Description	Inputs	Output
S1	System that allows the use of data belonging to the last two days for Bitcoin prediction	y(t-1), y(t-2)	y(t)
S2	System that allows the use of data belonging to the last three days for Bitcoin prediction	y(t-1), y(t-2), y(t-3)	y(t)
S3	System that allows the use of data belonging to the last four days for Bitcoin prediction	y(t-1), y(t-2), y(t-3), y(t-4)	y(t)

**Table 2:** Training results of the ANFIS training process using MPA to predict the Bitcoin price.

System	Number of MFs	Results			
		Best	Mean	Worst	Standard Deviation
S1	2	**7.8953** × 10^−4^	**9.4225** × 10^−4^	1.0228 × 10^−3^	6.10537 × 10^−5^
	3	8.8545 × 10^−4^	9.69855 × 10^−4^	1.0209 × 10^−3^	3.24048 × 10^−5^
	4	8.6929 × 10^−4^	9.60005 × 10^−4^	1.0195 × 10^−3^	3.86605 × 10^−5^
S2	2	8.3778 × 10^−4^	9.62309 × 10^−4^	1.0169 × 10^−3^	4.62243 × 10^−5^
	3	9.0084 × 10^−4^	9.69019 × 10^−4^	**1.0093** × 10^−3^	**2.91113 × 10^−5^**
S3	2	8.7446 × 10^−4^	9.652 × 10^−4^	1.0192 × 10^−3^	3.67125 × 10^−5^

**Table 3:** Testing results of the ANFIS training process using MPA to predict the Bitcoin price.

System	Number of MFs	Results			
		Best	Mean	Worst	Standard Deviation
S1	2	8.24916 × 10^−4^	**8.7879** × 10^−4^	**9.3408** × 10^−4^	2.73864 × 10^−5^
	3	8.58685 × 10^−4^	9.10183 × 10^−4^	9.94941 × 10^−4^	3.1039 × 10^−5^
	4	8.6031 × 10^−4^	9.06293 × 10^−4^	9.61275 × 10^−4^	2.836 × 10^−5^
S2	2	8.61192 × 10^−4^	9.0437 × 10^−4^	9.66542 × 10^−4^	**2.54936 × 10^−5^**
	3	8.6712 × 10^−4^	9.16834 × 10^−4^	1.14696 × 10^−3^	5.5415 × 10^−5^
S3	2	**7.66741** × 10^−4^	9.03833 × 10^−4^	1.16757 × 10^−3^	6.00471 × 10^−5^

**Table 4:** Comparison of the performances of some metaheuristic algorithms in ANFIS training for solving the relevant problem (best results are given in bold).

Algorithm	Train	Test
ABC	1.02805 × 10^−3^	9.64929 × 10^−4^
DA	1.09619 × 10^−3^	1.00396 × 10^−3^
FPA	1.03524 × 10^−3^	9.63824 × 10^−4^
MVO	1.0072 × 10^−3^	9.04124 × 10^−4^
RIME	9.96568 × 10^−4^	8.96077 × 10^−4^
TLBO	9.71073 × 10^−4^	8.8361 × 10^−4^
MPA (Proposed)	**9.4225** × 10^−4^	**8.7279** × 10^−4^

**Table 5:** Training results of the hybrid method based on FFNN and MPA to predict the Bitcoin price.

System	Network Structure	Results			
		Best	Mean	Worst	Standard Deviation
S1	2-3-1	1.0264 × 10^−3^	1.05171 × 10^−3^	1.1254 × 10^−3^	2.44954 × 10^−5^
	2-6-1	1.0089 × 10^−3^	1.03718 × 10^−3^	1.0862 × 10^−3^	1.71929 × 10^−5^
	2-9-1	1.0009 × 10^−3^	1.03369 × 10^−3^	1.0692 × 10^−3^	1.79111 × 10^−5^
	2-12-1	1.0017 × 10^−3^	1.03075 × 10^−3^	1.0656 × 10^−3^	1.47026 × 10^−5^
	2-15-1	1.0007 × 10^−3^	1.02777 × 10^−3^	1.0556 × 10^−3^	1.27463 × 10^−5^
	2-18-1	9.9825 × 10^−4^	1.02756 × 10^−3^	1.0675 × 10^−3^	1.66601 × 10^−5^
	2-21-1	9.9694 × 10^−4^	1.03365 × 10^−3^	1.0836 × 10^−3^	1.72811 × 10^−5^
S2	3-3-1	1.0195 × 10^−3^	1.05797 × 10^−3^	1.123 × 10^−3^	2.87589 × 10^−5^
	3-6-1	9.9301 × 10^−4^	1.03704 × 10^−3^	1.0925 × 10^−3^	2.39586 × 10^−5^
	3-9-1	9.9454 × 10^−4^	1.02951 × 10^−3^	1.0796 × 10^−3^	1.86775 × 10^−5^
	3-12-1	9.7688 × 10^−4^	1.02736 × 10^−3^	1.0846 × 10^−3^	2.57675 × 10^−5^
	3-15-1	**9.6513** × 10^−4^	1.02592 × 10^−3^	1.0704 × 10^−3^	2.10571 × 10^−5^
	3-18-1	9.6854 × 10^−4^	1.02407 × 10^−3^	1.0681 × 10^−3^	2.20877 × 10^−5^
	3-21-1	9.8083 × 10^−4^	1.03281 × 10^−3^	1.0923 × 10^−3^	2.30359 × 10^−5^
S3	4-3-1	1.0087 × 10^−3^	1.05994 × 10^−3^	1.1185 × 10^−3^	2.99406 × 10^−5^
	4-6-1	9.9567 × 10^−4^	1.03719 × 10^−3^	1.0948 × 10^−3^	2.77559 × 10^−5^
	4-9-1	9.9649 × 10^−4^	1.03083 × 10^−3^	1.0805 × 10^−3^	1.85184 × 10^−5^
	4-12-1	9.9952 × 10^−4^	1.03062 × 10^−3^	1.0603 × 10^−3^	1.9404 × 10^−5^
	4-15-1	9.6898 × 10^−4^	1.02706 × 10^−3^	1.108 × 10^−3^	3.15294 × 10^−5^
	4-18-1	9.8982 × 10^−4^	1.03197 × 10^−3^	1.0611 × 10^−3^	1.94366 × 10^−5^
	4-21-1	9.8732 × 10^−4^	**1.02298** × 10^−3^	1.0727 × 10^−3^	2.2127 × 10^−5^

**Table 6:** Testing results of the hybrid method based on FFNN and MPA to predict the Bitcoin price.

System	Network Structure	Results			
		Best	Mean	Worst	Standard Deviation
S1	2-3-1	8.36323 × 10^−4^	9.09951 × 10^−4^	1.2366 × 10^−3^	9.23503 × 10^−5^
	2-6-1	8.13556 × 10^−4^	8.91754 × 10^−4^	1.08444 × 10^−3^	6.13657 × 10^−5^
	2-9-1	8.38268 × 10^−4^	8.92329 × 10^−4^	9.92458 × 10^−4^	4.31368 × 10^−5^
	2-12-1	8.28592 × 10^−4^	8.78375 × 10^−4^	9.67174 × 10^−4^	3.45661 × 10^−5^
	2-15-1	8.07842 × 10^−4^	**8.72483** × 10^−4^	9.61714 × 10^−4^	4.03584 × 10^−5^
	2-18-1	8.28123 × 10^−4^	8.90745 × 10^−4^	1.05677 × 10^−3^	5.26151 × 10^−5^
	2-21-1	8.25916 × 10^−4^	8.92784 × 10^−4^	9.71171 × 10^−4^	3.83623 × 10^−5^
S2	3-3-1	8.29579 × 10^−4^	9.29238 × 10^−4^	1.13359 × 10^−3^	9.66076 × 10^−5^
	3-6-1	8.22897 × 10^−4^	8.99767 × 10^−4^	1.17899 × 10^−3^	7.23763 × 10^−5^
	3-9-1	8.17578 × 10^−4^	8.92761 × 10^−4^	9.99493 × 10^−4^	4.46318 × 10^−5^
	3-12-1	8.19318 × 10^−4^	8.73969 × 10^−4^	1.05125 × 10^−3^	4.75183 × 10^−5^
	3-15-1	8.21522 × 10^−4^	8.79057 × 10^−4^	1.00259 × 10^−3^	3.89623 × 10^−5^
	3-18-1	8.31353 × 10^−4^	8.94978 × 10^−4^	1.04906 × 10^−3^	4.76548 × 10^−5^
	3-21-1	**8.05415** × 10^−4^	8.94438 × 10^−4^	1.04307 × 10^−3^	4.92439 × 10^−5^
S3	4-3-1	8.34398 × 10^−4^	9.71803 × 10^−4^	1.17202 × 10^−3^	8.87335 × 10^−5^
	4-6-1	8.11659 × 10^−4^	9.20771 × 10^−4^	1.14152 × 10^−3^	7.65289 × 10^−5^
	4-9-1	8.29345 × 10^−4^	9.0755 × 10^−4^	1.00651 × 10^−3^	4.35021 × 10^−5^
	4-12-1	8.28965 × 10^−4^	9.124 × 10^−4^	1.0608 × 10^−3^	6.36791 × 10^−5^
	4-15-1	8.15115 × 10^−4^	9.09824 × 10^−4^	1.02788 × 10^−3^	5.46695 × 10^−5^
	4-18-1	8.48014 × 10^−4^	9.18519 × 10^−4^	1.0366 × 10^−3^	4.63586 × 10^−5^
	4-21-1	8.24326 × 10^−4^	8.95034 × 10^−4^	1.02614 × 10^−3^	4.15328 × 10^−5^

**Table 7:** Comparison of the performances of some metaheuristic algorithms in FFNN training for solving the relevant problem (best results are given in bold).

Algorithm	Train	Test
ABC	1.27154 × 10^−3^	1.23478 × 10^−3^
DA	2.35179 × 10^−3^	2.7986 × 10^−3^
FPA	1.25951 × 10^−3^	1.32675 × 10^−3^
MVO	1.1366 × 10^−3^	1.09176 × 10^−3^
RIME	1.2308 × 10^−3^	1.1989 × 10^−3^
TLBO	1.16626 × 10^−3^	1.0764 × 10^−3^
MPA (Proposed)	**1.02777** × 10^−3^	**8.72483** × 10^−4^

## Data Availability

The raw data supporting the conclusions of this article will be made available by the authors on request.

## Abbreviations

The following abbreviations are used in this manuscript:

**Abbreviation**	**Explanation**
ANFIS	Adaptive neuro-fuzzy inference system
ANN	Artificial neural network
FFNN	Feed-forward neural network
MPA	Marine predators algorithm
ABC	Artificial bee colony algorithm
FPA	Flower pollination algorithm
MVO	Multi-verse optimizer
DA	Dragonfly algorithm
TLBO	Teaching–learning-based optimization
RIME	RIME algorithm
MF	Membership function
gbellmf	Generalized bell-shaped membership function
MSE	Mean squared error
MAE	Mean absolute error
RMSE	Root mean squared error
MAPE	Mean absolute percentage error
PSO	Particle swarm optimization
GA	Genetic algorithm
MLP	Multi-layer perceptron
LSTM	Long short-term memory
RNNs	Recurrent neural networks
BPNN	Backpropagation neural network
